# Repertoire of Bovine miRNA and miRNA-Like Small Regulatory RNAs Expressed upon Viral Infection

**DOI:** 10.1371/journal.pone.0006349

**Published:** 2009-07-27

**Authors:** Evgeny A. Glazov, Kritaya Kongsuwan, Wanchai Assavalapsakul, Paul F. Horwood, Neena Mitter, Timothy J. Mahony

**Affiliations:** 1 Diamantina Institute for Cancer, Immunology and Metabolic Medicine, The University of Queensland, Princess Alexandra Hospital, Woolloongabba, Queensland, Australia; 2 CSIRO Livestock Industries, Queensland Bioscience Precinct, St Lucia, Queensland, Australia; 3 Department of Microbiology, Faculty of Science, Chulalongkorn University, Phayathai, Bangkok, Thailand; 4 Department of Primary Industries and Fisheries, Ritchie Building, Brisbane, Queensland, Australia; 5 School of Veterinary Sciences, University of Queensland, St Lucia, Queensland, Australia; Yale University, United States of America

## Abstract

MicroRNA (miRNA) and other types of small regulatory RNAs play a crucial role in the regulation of gene expression in eukaryotes. Several distinct classes of small regulatory RNAs have been discovered in recent years. To extend the repertoire of small RNAs characterized in mammals and to examine relationship between host miRNA expression and viral infection we used Illumina's ultrahigh throughput sequencing approach. We sequenced three small RNA libraries prepared from cell line derived from the adult bovine kidney under normal conditions and upon infection of the cell line with *Bovine herpesvirus* 1. We used a bioinformatics approach to distinguish authentic mature miRNA sequences from other classes of small RNAs and short RNA fragments represented in the sequencing data. Using this approach we detected 219 out of 356 known bovine miRNAs and 115 respective miRNA* sequences. In addition we identified five new bovine orthologs of known mammalian miRNAs and discovered 268 new cow miRNAs many of which are not identifiable in other mammalian genomes and thus might be specific to the ruminant lineage. In addition we found seven new bovine mirtron candidates. We also discovered 10 small nucleolar RNA (snoRNA) loci that give rise to small RNA with possible miRNA-like function. Results presented in this study extend our knowledge of the biology and evolution of small regulatory RNAs in mammals and illuminate mechanisms of small RNA biogenesis and function. New miRNA sequences and the original sequencing data have been submitted to miRNA repository (miRBase) and NCBI GEO archive respectively. We envisage that these resources will facilitate functional annotation of the bovine genome and promote further functional and comparative genomics studies of small regulatory RNA in mammals.

## Introduction

MicroRNAs (miRNAs) are small 21–23 nucleotide regulatory RNAs that modulate gene expression in animals and plants. In animals regulation of gene expression by miRNAs is achieved by sequence-specific targeting of the 3′ untranslated regions of messenger RNAs by the RNA induced silencing complex (RISC). This results in translational repression of protein synthesis and, in some cases, destabilization of messenger RNA [1]. The number of newly discovered miRNAs is growing rapidly [2], [3]. Moreover, several other classes of small regulatory RNAs, distinguished by their origin and biological functions, have been identified in recent years (for review see [4], [5]). These include small interfering RNAs (siRNAs), encompassing trans-acting siRNAs (tasiRNAs) and natural antisense transcript derived siRNAs (natsiRNAs), repeat-associated siRNAs (rasiRNAs, also referred to as PIWI-interacting RNAs or piRNAs), a recently identified group of snoRNA-derived miRNAs, and small RNAs associated with gene promoters (PASRs/tiRNAs) and 3′ termini (TASRs) [4]–[9].

Identification of comprehensive sets of miRNAs and other small regulatory RNAs in different organisms is a critical step to facilitate our understanding of genome organization, genome biology and evolution. The recently completed bovine genome is the first sequenced genome of ruminant mammal that displays a broad range of phenotypic characteristics that reflect adaptation to different habitats and domestication. The large number of cattle breeds selected for their commercially valuable traits (e.g. meat and milk production) and their ability to thrive in different environments make the bovine genome an attractive model to study genetic and epigenetics variations underlying diverse cattle phenotypes [10].

The latest release of the miRNA database (miRBase 13.0 March 2009) contains 356 bovine miRNA genes that code for 326 distinct mature miRNAs and eight sequences originating from the RNA hairpin arm opposite to the annotated mature miRNA containing arm, the so-called miRNA* [2]. Majority of these miRNAs have been identified based only on sequence similarity to known vertebrate miRNA orthologs and have never been confirmed experimentally [3], [11]. There were only two small scale studies describing cloning and experimental validation of novel miRNAs in cow [12], [13]. Furthermore, the total number of bovine miRNA genes is currently lower than that identified in other mammalian genomes such as mouse (547 miRNA genes) and human (706 miRNA genes). This suggests that there are still many undiscovered miRNAs in the bovine genome.

We aimed to extend the known repertoire of small regulatory RNAs expressed in bovine tissues and identify miRNA that could be disregulated upon viral infection. We used a cell line derived from the adult bovine kidney under normal conditions and upon infection of the cell line with the *Bovine herpesvirus* 1 (BoHV-1). Illumina's ultrahigh throughput sequencing approach has been used successfully to identify new miRNAs and other classes of small regulatory RNAs in several recent studies [6], [9], [14]–[18]. We utilized this approach to sequence and analyze three small RNA libraries. One library was prepared from mock-infected cells and two others prepared from cells infected with BoHV-1 at two different multiplications of infectivity (MOI). Each library was sequenced individually and generated approximately five million short sequence reads resulting in a total of almost 15 million sequence reads. We utilized a recently developed bioinformatics pipeline to distinguish authentic mature miRNA sequences from other small RNAs and short RNA fragments represented in the sequencing data [15]. Here, we describe a detailed analysis of this sequence data and its interpretation.

## Results and Discussion

To simplify the sequencing data, all identical sequence reads in each small RNA library were grouped and converted into sequence tags–unique sequences with associated counts of the individual sequence reads. The resulting sets of non-redundant sequence tags for each library were mapped to the bovine and BoHV-1 reference genomes [10]. We found that the vast majority of the mapped sequence tags originated from the bovine genome and only a small fraction was derived from the BoHV-1 genome. A detailed analysis and characterization of the small RNA originating from the viral genome has been described elsewhere (manuscript submitted).

### Known bovine miRNAs and bovine orthologs of known mammalian miRNAs

Following mapping of sequence tags to the reference bovine genome we analyzed small RNA sequences originating from known bovine miRNA loci listed in miRBase 13.0. We found that out of 356 known cow miRNAs 338 could be positioned onto assembled chromosomes; out of those 219 miRNA were detected in at least one of our three small RNA libraries, 190 amongst those were detected in either two or all three small RNA libraries (Supplemental Table S1). We also identified sequence tags representing miRNA* sequences for 115 out of 219 expressed miRNA genes, a large increase over the eight reported previously (Supplemental Table S1). This finding is consistent with several deep sequencing studies performed in other vertebrates that illustrated higher sensitivity of deep sequencing approach over methods such as Northern hybridization and microarrays [15], [18], [19]. In five cases (bta-mir-30b, bta-mir-193a, bta-mir-345, bta-mir-365, bta-mir-423) we discovered that miRNA* are more abundant than corresponding miRNA as evidenced by higher counts of sequence reads originating from miRNA* arms of the microRNA precursor sequences (Supplemental Table S1). Although these cases could be simple annotation artifacts, it is also possible that they reflect regulated processing of pre-miRNA that results in preferential utilization of different arms of miRNA precursor [15], [20]. We also identified two miRNAs (bta-mir-23b and bta-mir-27a) that demonstrated nearly equal number of sequence reads originating from the 5′ and 3′ arms of the miRNA hairpin precursor (Supplemental Table S1). This type of expression pattern is representative of miRNA genes that have similar 5′ end stability of the processed small RNA duplex that leads to equal incorporation of either strand into the RISC and their protection from degradation [20]. A few such miRNA genes have been predicted and validated in different species [15], [21], [22].

As reflected by the total counts of miRNA-derived sequence reads, known miRNAs had a very broad range of expression which varied from thousands sequence reads for the most abundant miRNAs such as ubiquitous let-7 miRNA family, bta-mir-21, and bta-mir-140 to zero for the approximately one quarter of known cow miRNAs that have not been detected in our small RNA libraries (Supplemental Table S1).

Several previous studies have demonstrated that many vertebrate miRNA genes show high level of evolutionary conservation [23]–[27]. Given that number of known cow miRNAs in miRBase is substantially smaller than that identified in other mammalian genomes such as mouse and human we reasoned that at least some of the miRNA identified in other mammals would have orthologous evolutionary conserved sequences present in bovine genome (Supplemental Figure S1). To validate this hypothesis we used non-redundant set of known human, dog and mouse miRNAs sequences listed in miRBase 13.0 to search for highly similar DNA sequences in the bovine genome. Sequences returned by sequence similarity searches were then confirmed as orthologous miRNA candidates by analysis of their predicted RNA structures (File S1). Using this approach we identified five new orthologous miRNAs in cattle genome (cfa-mir-1842, cfa-mir-194, hsa-mir-1277, hsa-mir-1468, hsa-mir-320a) that were expressed in our deep sequencing data; two of those also had detectable miRNA* (cfa-mir-1842*, cfa-mir-194*) (Supplemental Table S1). The relatively small number of the newly discovered bovine orthologs is consistent with emerging notion that the majority of highly evolutionary conserved miRNA that are present within the entire mammalian lineage has already been identified. Most of the miRNA discovered recently appear to be present only within narrow phylogenetic groups [25], [28], [29].

In an earlier miRNA discovery study we observed rare cases suggestive of unusual miRNA processing as evidenced by uncharacteristic distribution of small RNA sequence tags mapped to miRNA loci [15]. These loci had detectable small RNA sequence tags originating from either the terminal loop of the miRNA precursor or from its distal ends. Interestingly, two miRNA reported in the previous study in chicken (gga-mir-451 and gga-mir-218) also had detectable small sequence tags originating from terminal loop regions in the current study (Supplemental Figures S2a, S2b). Although similar examples have also been reported in other studies they were usually regarded as miRNA processing intermediates detected due to the much larger volume of sequence data [29], [30]. This is probably true for most of such cases, however, unusually high proportion of sequence tags originating from terminal loop region relative to abundance of known mature miRNA, and detection of similar pattern in two distantly related organisms suggests that at least in some cases such as mir-451 and miRNA-218 small RNA sequences derived from terminal loop region of the miRNA precursor may be functionally active [15]. Another example of unusual miRNA processing presented in the Supplemental Figure S2c shows small RNA sequence tags originating from distal ends of the bovine miRNA bta-mir-21 precursor. Remarkably, this distribution of small RNA tags strongly resembles pattern of endogenous siRNA processing recently discovered in fruit fly and mouse suggesting possible cross-talk between the two pathways [16], [31].

### Bidirectionaly transcribed miRNAs

**Figure 1:**
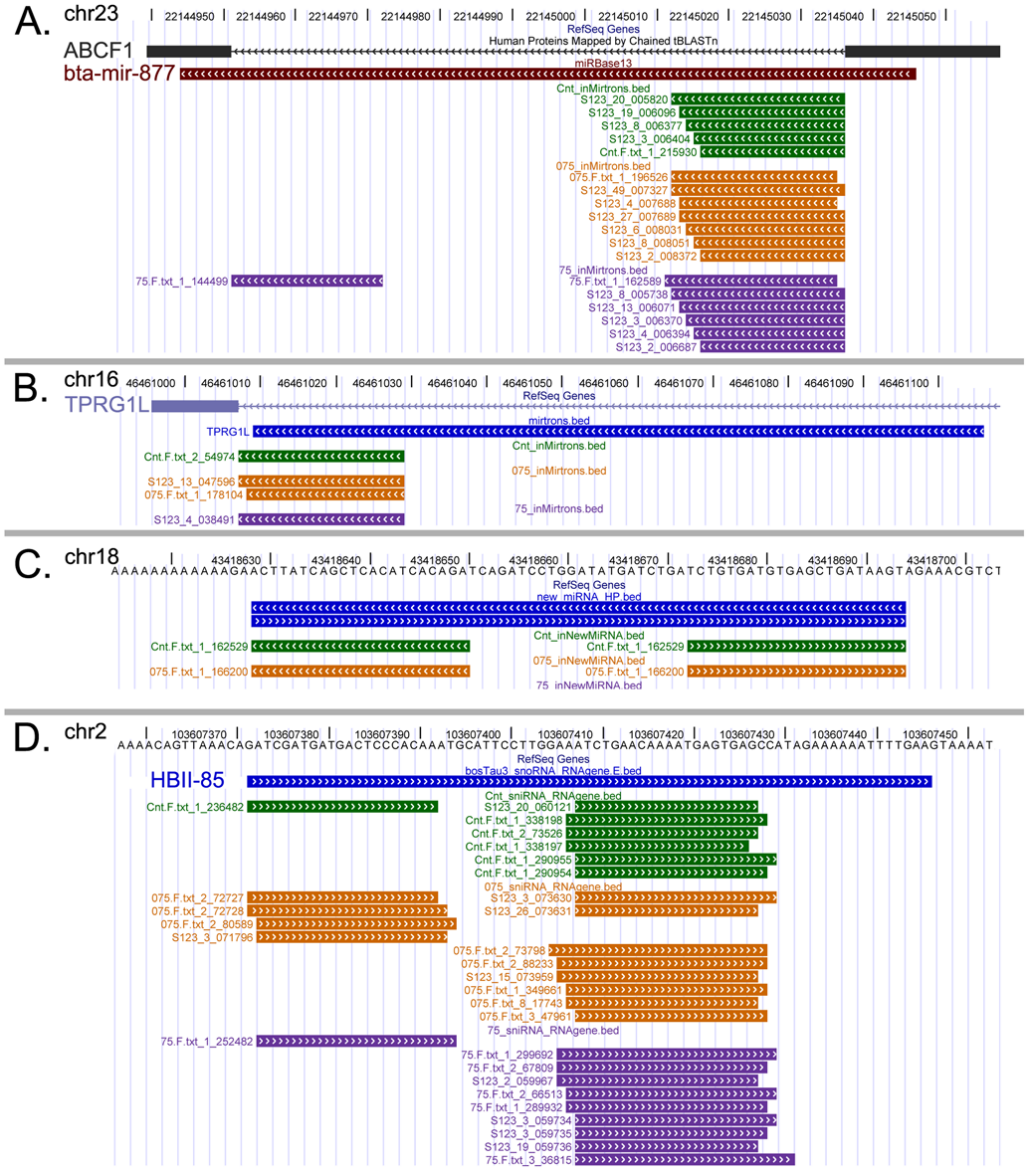
Small regulatory RNA in the bovine genome. Each panel shows UCSC genome browser screens. Small RNA sequence tags originating from the three small RNA libraries are shown as follows: mock-infected control - green, MOI 0.75 library - dark orange, MOI 7.5 library–magenta. Predicted pre-miRNA hairpin-like precursors are shown in blue. Arrowheads indicate alignment of sequences relative to the genomic strands. (A) Evolutionary conserved mirtron bta-mir-877 (deep red) located within intron of the *ABCF1* gene. *ABCF1* exon-intron junction is shown as black block (exon) and thin black line (intron) (B) New tailed mirtron located within intron of the *TPRG1L* gene. *TPRG1L* exon-intron junction is shown as light blue block (exon) and thin light blue line (intron) (C) New bovine miRNA with predicted miRNA precursors (blue) on both genomic strands and small RNAs originating from positive (right) and negative (left) genomic strands. (D) Small RNA sequence tags originating from an orphan snoRNA HBII-85 locus (blue). The distribution of small RNA tags closely resembles pattern characteristic of Dicer-dependent processing.

Three recent studies in fruit fly and other organisms reported cases of miRNA loci characterized by bidirectional transcription from both genomic DNA strands that gives rise to functionally distinct miRNAs [32]–[34]. Inspired by these findings we investigated presence of such miRNA loci in cow genome. Although, mammalian miRNA mir-338 reported by Tyler and colleagues didn't show any evidence of bidirectional transcription in our experimental system (data not shown), we identified four new loci showing evidence of bidirectional transcription amongst known bovine miRNA and additional five loci amongst newly identified bovine miRNA (Figure 1C, Supplemental Figures S3a–S3h). In another case (bta-mir-219, Supplemental Figure S6) we found that the majority of the small RNA sequence tags were derived from the genomic strand opposite to the currently annotated miRNA. Although, it is likely that this case is a simple miRNA annotation error it is also possible that bta-mir-219 could display regulated bi-directional transcription similar to the examples described above.

### Newly identified miRNAs

To identify novel miRNAs in the sequencing data from the three small RNA libraries we used the following criteria: (1) genomic loci annotated as known bovine miRNAs or as other classes of non-coding RNA were excluded; (2) to be considered for further analysis an individual locus had to be supported by at least two independent sequence reads originating from at least two small RNA libraries; (3) the loci lacking hairpin-like RNA secondary structures including the positions of the small RNA tags were eliminated. The resulting set of sequences and their respective RNA structures were analyzed further to distinguish genuine miRNA precursors from other RNAs that contain similar RNA structures (e.g. tRNA-derived repeat elements; File S1).

The resulting dataset was comprised of 268 unique sequences identified as novel bovine miRNA and miRNA* (Supplemental Tables S3–S5). Analysis of evolutionary conservation of the newly identified bovine miRNAs in the human, mouse, dog, horse, and possum genome assemblies didn't reveal any new conserved miRNA except the five new bovine orthologs of known mammalian miRNAs identified previously. This result suggests that majority of the newly identified miRNA might be specific to the ruminant lineage.

### Mirtrons

Several recent studies have described an alternative miRNA processing pathway which uses intron splicing machinery instead of the Drosha endonuclease to generate miRNA precursors from intronic sequences [15], [35]–[38]. A distinct feature of such miRNA-generating introns is that the miRNA hairpin-like precursor is directly adjacent to the splice sites such that mature miRNA sequences often start directly at the 5′ terminus of the intron and/or end at its 3′ terminus. The few mirtrons identified to date originate from the diverse evolutionarily lineages of insect (*Drosophila sp.*), worms (*Caenorhabditis elegans*), and mammals (primates and rodents) [15], [35]–[38]. Identification of mirtrons in sequencing data is heavily reliant on comprehensive annotations on genes and exon-intron junctions [15], [37], [38]. This is why bioinformatics approaches used in other well annotated genomes have only a limited applicability in cattle due to the present under-annotation of the bovine genome. Considering these factors we used a combination of bioinformatics and comparative genomics approaches to search for mirtrons in bovine genome. For *de-novo* identification of mirtrons we used methodology described earlier [15]. This analysis yielded two tailed-mirtrons - these type of mirtrons do not span the entire length of introns but are directly adjacent to either donor or acceptor splice sites and were previously referred to as “atypical mirtrons” [15], [38]. These mirtrons were localised at the acceptor splice sites within *TPRG1L* and *DDX5* genes respectively and were expressed in all three small RNA libraries as evidenced by presence of sequence tags (Figure 1B, Supplemental Figure S4a). The example of the *DDX5* mirtron illustrates well present challenge of identification of mirtrons in the bovine genome–there is no RefSeq annotation currently available for the bovine *DDX5* gene (Supplemental Figure S4a), and as a result *DDX5* mirtron could only be identified using human protein annotations mapped onto bovine genome (Supplemental Figure S4a). Therefore we recognize that some bovine-specific mirtrons would have been missed in our analysis due to the lack of comprehensive annotations of the bovine genes. For comparative genomics analyses we used known mammalian mirtrons identified in two recent studies [37], [38]. Although we were able to identify five sequences that were evolutionary conserved in terms of sequence similarity, microsynteny between genomes, and predicted RNA secondary structure, only one of them had evidence of expression in our sequencing libraries (Figure 1A, Supplemental Figures S4b–S4e). This mirtron known as mir-877 is localised within *ABCF1* gene and has been also detected in human, chimpanzee, mouse and rat in two recent studies (Figure 1A) [37], [38].

### snoRNA-derived small RNAs

Small nucleolar RNA (snoRNA) is a class of evolutionary conserved non-coding RNAs present throughout the Eukaryotes [39]. Their best characterized function is catalysis of maturation of spliceosomal and ribosomal RNA by guiding methylation and pseudouridylation of target RNAs in sequence-specific manner [39]. Three recent studies including ours, uncovered an unexpected link between snoRNA and small regulatory RNA biogenesis pathways [6], [8], [40]. These studies demonstrated that snoRNA could be further processed into small RNA and that this processing requires AGO and DICER–two essential enzymes involved in miRNA and siRNA processing [4], [6], [8], [41].

Although analysis of snoRNA loci in the bovine genome is complicated by the current lack of comprehensive annotation of non-coding RNAs we analyzed bovine snoRNA loci that could be identified using human snoRNA annotations and syntenic alignment tool from UCSC genome browser (File S1). We identified ten snoRNA genes and two snRNA genes that gave rise to small RNAs with typical miRNA and/or siRNA characteristics (Figure 1D, Supplemental Figures S5a–S5k). Identification of small RNA originating from HBII-85 snoRNA is particularly intriguing (Figure 1D). This snoRNA was originally identified in mouse where it was found to be expressed predominantly in brain, and to the lesser extent in muscle, kidney, and lung [42]. Together with other snoRNA lacking obvious targets within spliceosomal and/or ribosomal RNA this snoRNA was classified as “orphan” [43]. Although a possible link between HBII-85 and Prader–Willi syndrome was suggested at the time of discovery its definite role in the disease was confirmed only recently by two genetic studies in mouse and human [42], [44], [45]. Nevertheless, the molecular mechanisms behind HBII-85 function still remain to be fully elucidated. Given the two recent studies that demonstrated that snoRNA can serve as miRNA precursor [6], [8], it is tempting to suggest that snoRNA HBII-85 and other similar “orphan” snoRNA function predominantly as processed small RNA species in miRNA and/or siRNA pathways [4].

## Materials and Methods

### Construction of small RNA libraries and ultrahigh throughput sequencing

The source of bovine RNA, RNA sample preparation, and construction of small RNA sequencing libraries is described in supplemental methods (File S1). In brief, a cultured MDBK cell line derived from normal adult bovine kidney was used a source of bovine RNA [46]. The MDBK monolayers were either mock inoculated or infected with the infection clone pBACBHV-37 [47], of the BoHV-1, strain V155 at multiplications of infectivity (MOI) of 0.75 and 7.5. At 6 h post-infection monolayers were harvested and total RNA isolated using Trizol reagent (Invitrogen) according to the manufacturer's instructions.

Approximately 20 µg of total RNA isolated from MDBK cells either infected (MOI of 0.75 and 7.5) or mock inoculated with BoHV-1 was supplied to GeneWorks Pty Ltd (Adelaide, Australia) for construction of small RNA libraries and ultrahigh throughput sequencing. Sequencing was performed on the Illumina Genome Analyzer G1 according to the manufacturer's protocol.

### Analysis of sequencing data

Individual sequence reads with the base quality scores were produced by GeneWorks Pty Ltd using Illimina's Data Analysis Pipeline software v.1.0. Subsequent sequence data analyses were carried out as described by Glazov et al. with some modification [15]. All identical sequences were counted and combined into one record. The resulting set of the unique sequences with associated ‘read counts’ is referred to as sequence tags. A mirror of the UCSC genome browser and database was created with the *Bos Taurus v. 3* genome sequence assembly and annotations (bosTau3, August 2006) [48], [49]. After trimming the 3′ adaptor sequence, sequence tags were mapped onto bovine and BoHV-1 (GenBank AJ004801) genome assemblies using BLAT software [50]. To identify sequence tags originating from coding exons, repeats, rRNA, tRNA, snRNA and snoRNA we used UCSC “RefGene”, “RepeatMasker” and NCBI “RefSeq” data [48], [51], as well as our own sets of ncRNA annotations compiled from the NCBI GenBank data (http://www.ncbi.nlm.nih.gov/). To identify novel miRNA genes we identified all hairpin-like RNA structures encompassing small RNA sequence tags using RNAfold [52]; then we analyzed sequence and structural features of the predicted hairpin-like structures to distinguish genuine miRNA precursors from other RNA classes that may contain similar RNA structures (e.g. tRNA-derived repeat).

To identify the evolutionary conserved orthologs of the bovine miRNAs in other mammalian genomes we used bovine pre-miRNA sequences to search for highly similar sequences in human, mouse, dog, horse, and opossum genomes. The search was performed using BLAT Kent 2002 [50] with the following parameters: -noHead -minMatch = 1 -oneOff = 1 -minIdentity = 90 -tileSize = 8. Sequence alignments covering at least 80% of the length of BoHV-1 pre-miRNA were considered as potential orthologs and used in further RNA secondary structure analyses.

Known mammalian miRNA sequences were obtained from microRNA database at the Sanger center (miRBase 13.0 March 2009) [3].

### Data deposition and accession numbers

All sequences identified as new miRNA precursors and the mature miRNA were submitted to the miRBase at the Sanger Centre (http://microrna.sanger.ac.uk/registry/). Original sequence data generated in this study have been deposited to the NCBI Gene Expression Omnibus database (http://www.ncbi.nlm.nih.gov/geo/) under accession number GSE15450.

## Supporting Information

Table S1 Summary of the expression data for known detected cow miRNA
(0.16 MB XLS)

Table S2 Sequence and genomic coordinates information for new miRNA* identified in this study.
(0.03 MB XLS)

Table S3 Summary of the expression data for newly detected cow miRNA
(0.06 MB XLS)

Table S4 Sequence and genomic coordinates information for new miRNA precursors
(0.03 MB XLS)

Table S5 Sequence and genomic coordinates information for new mature miRNA and miRNA*
(0.03 MB XLS)

Figure S1 Summary of phylogenetic relationships of the mammalian species discussed in this study.
(0.06 MB PDF)

Figure S2 Known miRNA with unusual small RNA processing pattern.
(0.10 MB PDF)

Figure S3 Bidirectional miRNA candidates.
(0.10 MB PDF)

Figure S4 Mirtrons
(0.09 MB PDF)

Figure S5 snoRNA-derived small RNAs
(0.13 MB PDF)

Figure S6 Bovine miRNA bta-mir-219
(0.06 MB PDF)

File S1 Supplemental methods.
(0.03 MB PDF)

## References

[1] He L, Hannon GJ (2004). MicroRNAs: small RNAs with a big role in gene regulation.. Nat Rev Genet.

[2] Griffiths-Jones S, Grocock RJ, van Dongen S, Bateman A, Enright AJ (2006). miRBase: microRNA sequences, targets and gene nomenclature.. Nucleic Acids Research.

[3] Griffiths-Jones S, Saini HK, van Dongen S, Enright AJ (2008). miRBase: tools for microRNA genomics.. Nucleic Acids Res.

[4] Ghildiyal M, Zamore PD (2009). Small silencing RNAs: an expanding universe.. Nat Rev Genet.

[5] Chapman EJ, Carrington JC (2007). Specialization and evolution of endogenous small RNA pathways.. Nat Rev Genet.

[6] Ender C, Krek A, Friedlander MR, Beitzinger M, Weinmann L (2008). A human snoRNA with microRNA-like functions.. Mol Cell.

[7] Kapranov P, Cheng J, Dike S, Nix DA, Duttagupta R (2007). RNA maps reveal new RNA classes and a possible function for pervasive transcription.. Science.

[8] Saraiya AA, Wang CC (2008). snoRNA, a novel precursor of microRNA in Giardia lamblia.. PLoS Pathog.

[9] Taft RJ, Glazov EA, Cloonan N, Simons C, Stephen S (2009). Tiny RNAs associated with transcription start sites in animals.. Nat Genet.

[10] Elsik CG, Tellam RL, Worley KC, Gibbs RA, Muzny DM (2009). The Genome Sequence of Taurine Cattle: A Window to Ruminant Biology and Evolution.. Science.

[11] Strozzi F, Mazza R, Malinverni R, Williams JL (2009). Annotation of 390 bovine miRNA genes by sequence similarity with other species.. Anim Genet.

[12] Gu Z, Eleswarapu S, Jiang H (2007). Identification and characterization of microRNAs from the bovine adipose tissue and mammary gland.. FEBS Lett.

[13] Coutinho LL, Matukumalli LK, Sonstegard TS, Van Tassell CP, Gasbarre LC (2007). Discovery and profiling of bovine microRNAs from immune-related and embryonic tissues.. Physiol Genomics.

[14] Grimson A, Srivastava M, Fahey B, Woodcroft BJ, Chiang HR (2008). Early origins and evolution of microRNAs and Piwi-interacting RNAs in animals.. Nature.

[15] Glazov EA, Cottee PA, Barris WC, Moore RJ, Dalrymple BP (2008). A microRNA catalog of the developing chicken embryo identified by a deep sequencing approach.. Genome Res.

[16] Czech B, Malone CD, Zhou R, Stark A, Schlingeheyde C (2008). An endogenous small interfering RNA pathway in Drosophila.. Nature.

[17] Project AET (2009). Post-transcriptional processing generates a diversity of 5′-modified long and short RNAs.. Nature.

[18] Morin RD, O'Connor MD, Griffith M, Kuchenbauer F, Delaney A (2008). Application of massively parallel sequencing to microRNA profiling and discovery in human embryonic stem cells.. Genome Res.

[19] Marioni JC, Mason CE, Mane SM, Stephens M, Gilad Y (2008). RNA-seq: An assessment of technical reproducibility and comparison with gene expression arrays.. Genome Res.

[20] Winter J, Jung S, Keller S, Gregory RI, Diederichs S (2009). Many roads to maturity: microRNA biogenesis pathways and their regulation.. Nat Cell Biol.

[21] Schwarz DS, Hutvagner G, Du T, Xu Z, Aronin N (2003). Asymmetry in the assembly of the RNAi enzyme complex.. Cell.

[22] Aravin AA, Lagos-Quintana M, Yalcin A, Zavolan M, Marks D (2003). The small RNA profile during Drosophila melanogaster development.. Dev Cell.

[23] Glazov EA, McWilliam S, Barris WC, Dalrymple BP (2008). Origin, evolution, and biological role of miRNA cluster in DLK-DIO3 genomic region in placental mammals.. Mol Biol Evol.

[24] Berezikov E, Guryev V, van de Belt J, Wienholds E, Plasterk RH (2005). Phylogenetic shadowing and computational identification of human microRNA genes.. Cell.

[25] Bentwich I, Avniel A, Karov Y, Aharonov R, Gilad S (2005). Identification of hundreds of conserved and nonconserved human microRNAs.. Nat Genet.

[26] Altuvia Y, Landgraf P, Lithwick G, Elefant N, Pfeffer S (2005). Clustering and conservation patterns of human microRNAs.. Nucleic Acids Res.

[27] Hertel J, Lindemeyer M, Missal K, Fried C, Tanzer A (2006). The expansion of the metazoan microRNA repertoire.. BMC Genomics.

[28] Berezikov E, Thuemmler F, van Laake LW, Kondova I, Bontrop R (2006). Diversity of microRNAs in human and chimpanzee brain.. Nature Genetics.

[29] Ruby JG, Stark A, Johnston WK, Kellis M, Bartel DP (2007). Evolution, biogenesis, expression, and target predictions of a substantially expanded set of Drosophila microRNAs.. Genome Res.

[30] Ruby JG, Jan C, Player C, Axtell MJ, Lee W (2006). Large-scale sequencing reveals 21U-RNAs and additional microRNAs and endogenous siRNAs in C. elegans.. Cell.

[31] Watanabe T, Totoki Y, Toyoda A, Kaneda M, Kuramochi-Miyagawa S (2008). Endogenous siRNAs from naturally formed dsRNAs regulate transcripts in mouse oocytes.. Nature.

[32] Stark A, Bushati N, Jan CH, Kheradpour P, Hodges E (2008). A single Hox locus in Drosophila produces functional microRNAs from opposite DNA strands.. Genes Dev.

[33] Bender W (2008). MicroRNAs in the Drosophila bithorax complex.. Genes Dev.

[34] Tyler DM, Okamura K, Chung WJ, Hagen JW, Berezikov E (2008). Functionally distinct regulatory RNAs generated by bidirectional transcription and processing of microRNA loci.. Genes Dev.

[35] Ruby JG, Jan CH, Bartel DP (2007). Intronic microRNA precursors that bypass Drosha processing.. Nature.

[36] Okamura K, Hagen JW, Duan H, Tyler DM, Lai EC (2007). The mirtron pathway generates microRNA-class regulatory RNAs in Drosophila.. Cell.

[37] Berezikov E, Chung WJ, Willis J, Cuppen E, Lai EC (2007). Mammalian mirtron genes.. Mol Cell.

[38] Babiarz JE, Ruby JG, Wang Y, Bartel DP, Blelloch R (2008). Mouse ES cells express endogenous shRNAs, siRNAs, and other Microprocessor-independent, Dicer-dependent small RNAs.. Genes Dev.

[39] Matera AG, Terns RM, Terns MP (2007). Non-coding RNAs: lessons from the small nuclear and small nucleolar RNAs.. Nat Rev Mol Cell Biol.

[40] Taft RJ, Glazov EA, Lassmann T, Hayashizaki Y, Carninci P (2009). Small RNAs derived from snoRNAs.. RNA.

[41] Hutvagner G, Simard MJ (2008). Argonaute proteins: key players in RNA silencing.. Nat Rev Mol Cell Biol.

[42] Cavaille J, Buiting K, Kiefmann M, Lalande M, Brannan CI (2000). Identification of brain-specific and imprinted small nucleolar RNA genes exhibiting an unusual genomic organization.. Proceedings of the National Academy of Sciences of the United States of America.

[43] Bachellerie JP, Cavaille J, Huttenhofer A (2002). The expanding snoRNA world.. Biochimie.

[44] Skryabin BV, Gubar LV, Seeger B, Pfeiffer J, Handel S (2007). Deletion of the MBII-85 snoRNA gene cluster in mice results in postnatal growth retardation.. PLoS Genet.

[45] Sahoo T, del Gaudio D, German JR, Shinawi M, Peters SU (2008). Prader-Willi phenotype caused by paternal deficiency for the HBII-85 C/D box small nucleolar RNA cluster.. Nat Genet.

[46] Madin SH, Darby NB (1958). Established kidney cell lines of normal adult bovine and ovine origin.. Proc Soc Exp Biol Med.

[47] Mahony TJ, McCarthy FM, Gravel JL, West L, Young PL (2002). Construction and manipulation of an infectious clone of the bovine herpesvirus 1 genome maintained as a bacterial artificial chromosome.. J Virol.

[48] Karolchik D, Kuhn RM, Baertsch R, Barber GP, Clawson H (2008). The UCSC Genome Browser Database: 2008 update.. Nucleic Acids Res.

[49] Kent WJ, Sugnet CW, Furey TS, Roskin KM, Pringle TH (2002). The human genome browser at UCSC.. Genome Research.

[50] Kent WJ (2002). BLAT–the BLAST-like alignment tool.. Genome Res.

[51] Kuhn RM, Karolchik D, Zweig AS, Trumbower H, Thomas DJ (2007). The UCSC genome browser database: update 2007.. Nucleic Acids Res.

[52] Hofacker IL (2003). Vienna RNA secondary structure server.. Nucleic Acids Res.

