# Supplementary material for: Repertoire of Bovine miRNA and miRNA-Like Small Regulatory RNAs Expressed upon Viral Infection
Source: PLoS One. 2009 Jul 27;4(7):e6349. doi: 10.1371/journal.pone.0006349 (PMC2713767; doi:10.1371/journal.pone.0006349)
Supplement: Figure S1 — Summary of phylogenetic relationships of the mammalian species discussed in this study. (0.06 MB PDF) [file pone.0006349.s006.pdf]

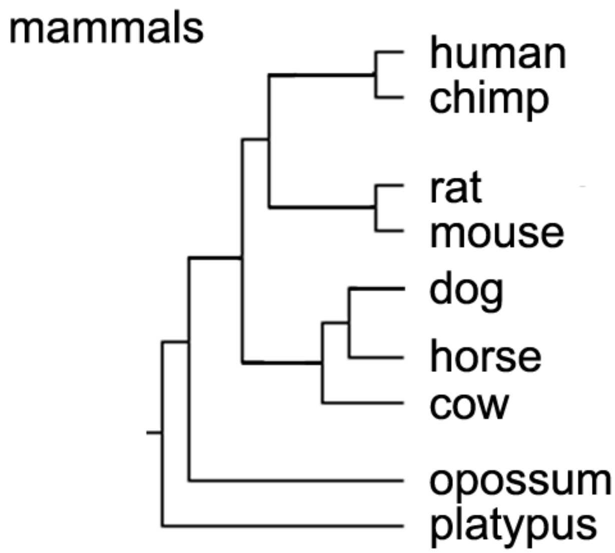

Supplemental Figure S1. Summary of phylogenetic relationships of the mammalian species discussed in this study. The tree structure used with modifications from Hedges et al. (Hedges et al. 2006).
