# Supplementary material for: Repertoire of Bovine miRNA and miRNA-Like Small Regulatory RNAs Expressed upon Viral Infection
Source: PLoS One. 2009 Jul 27;4(7):e6349. doi: 10.1371/journal.pone.0006349 (PMC2713767; doi:10.1371/journal.pone.0006349)
Supplement: Figure S2 — Known miRNA with unusual small RNA processing pattern. (0.10 MB PDF) [file pone.0006349.s007.pdf]

Glazov EA et al. Supplemental Figure S2

Fig S2a.

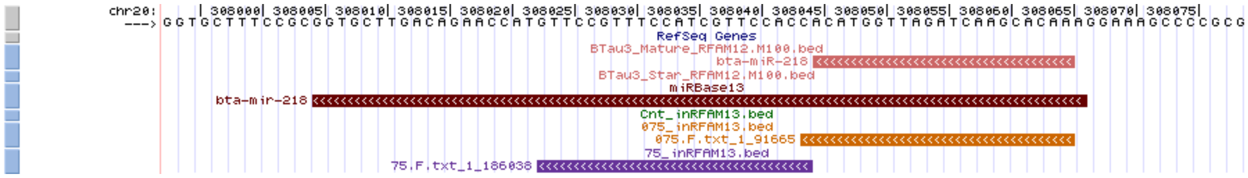

Fig S2b.

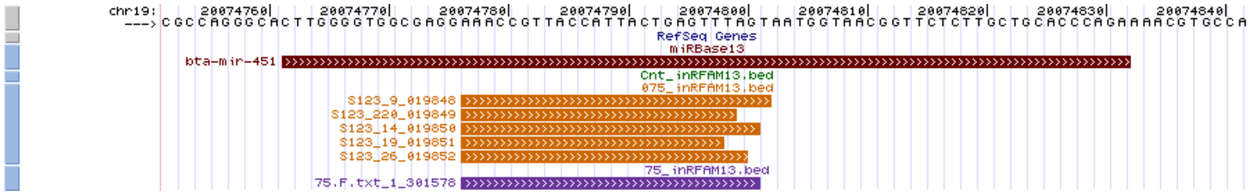

Glazov EA et al. Supplemental Figure S2

Fig S2c.

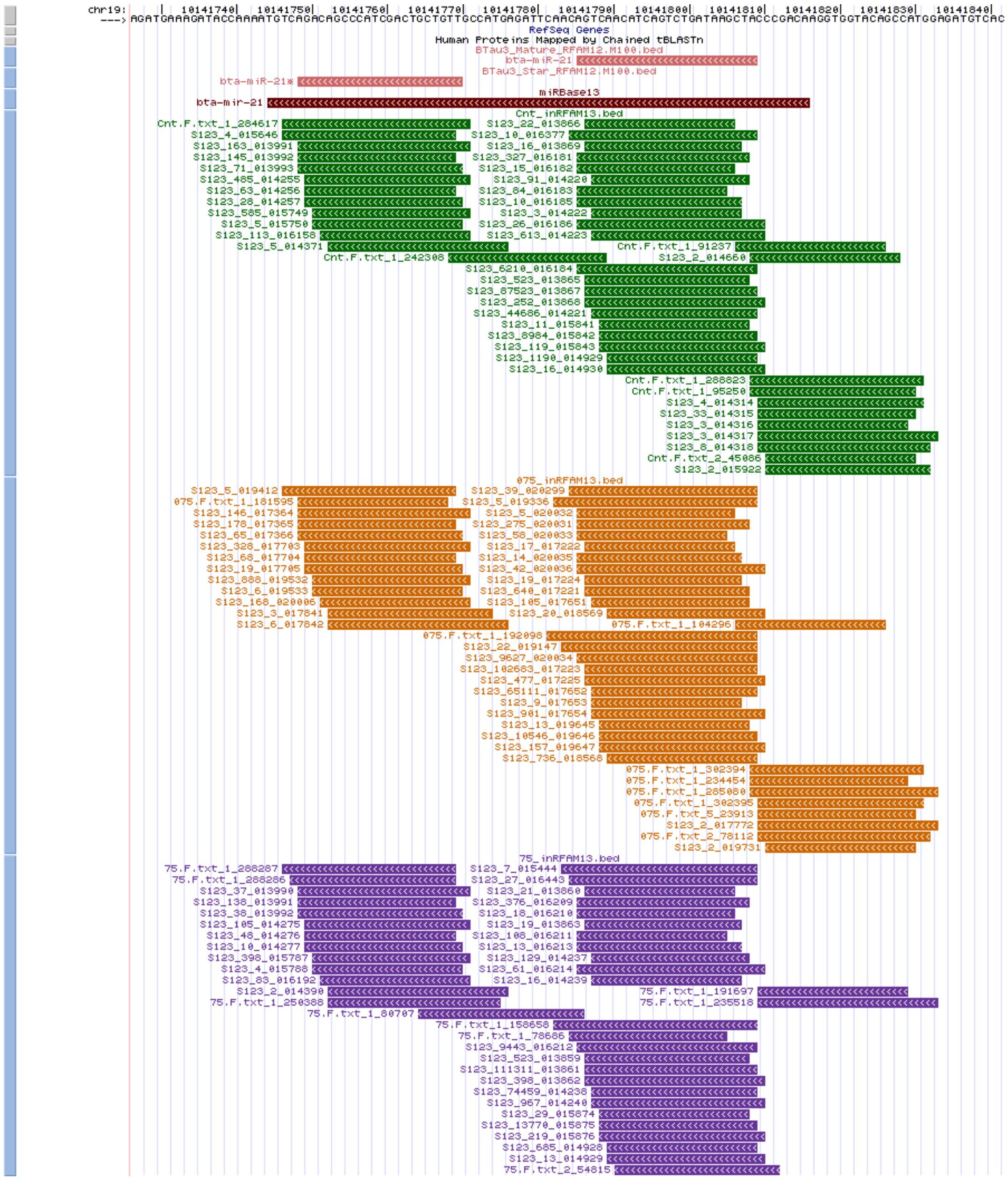

Supplemental Figures S2a-S2c. Known miRNA with unusual small RNA processing pattern. (S2a, S2b) bta-mir-218 and bta-mir-451 have detectable small RNA sequence tags originating from the terminal loop region of the pre-miRNA precursor. **(S2c)** bta-mir-21 has detectable small RNA sequence tags derived from distal ends of the pre-miRNA precursor. The figures shows UCSC genome browser screens displaying relative positions of predicted miRNA hairpin-like precursors (blue) or known miRNA precursor (deep red), and sequence tags originating from this loci in three small RNA libraries: mock-infected control - green, MOI 0.75 library - dark orange, MOI 7.5 library – magenta. Arrowheads indicate alignment of sequences relative to the genomic strands.
