# Supplementary material for: Repertoire of Bovine miRNA and miRNA-Like Small Regulatory RNAs Expressed upon Viral Infection
Source: PLoS One. 2009 Jul 27;4(7):e6349. doi: 10.1371/journal.pone.0006349 (PMC2713767; doi:10.1371/journal.pone.0006349)
Supplement: Figure S3 — Bidirectional miRNA candidates. (0.10 MB PDF) [file pone.0006349.s008.pdf]

Glazov EA et al. Supplemental Figure S3

Fig S3a.

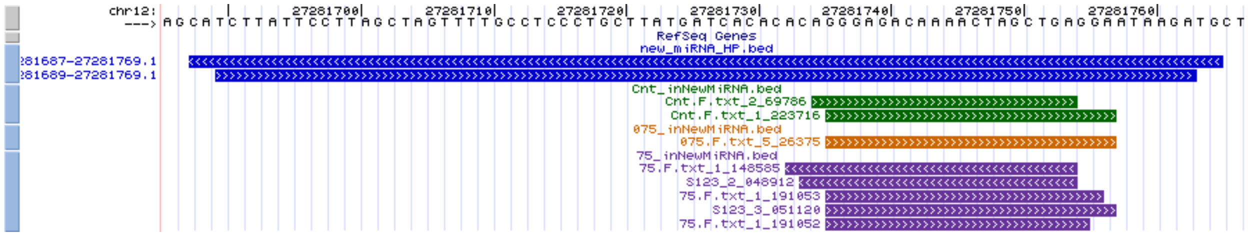

Fig S3b.

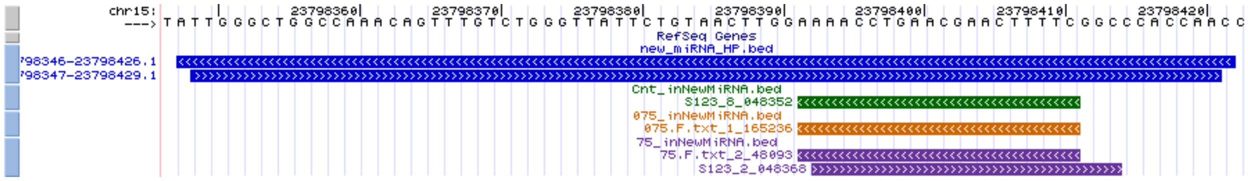

Fig S3c.

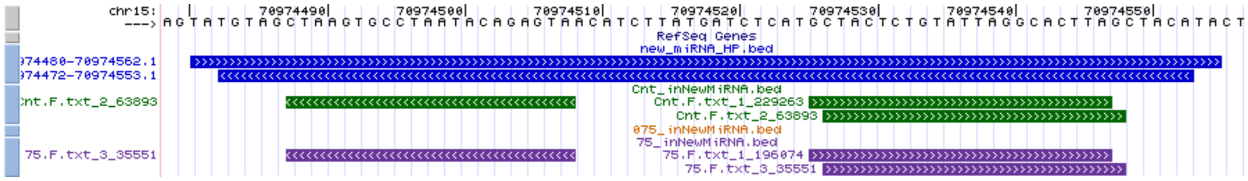

Fig S3d.

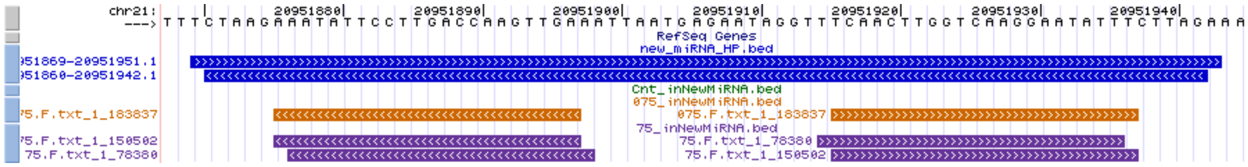

Fig S3e.

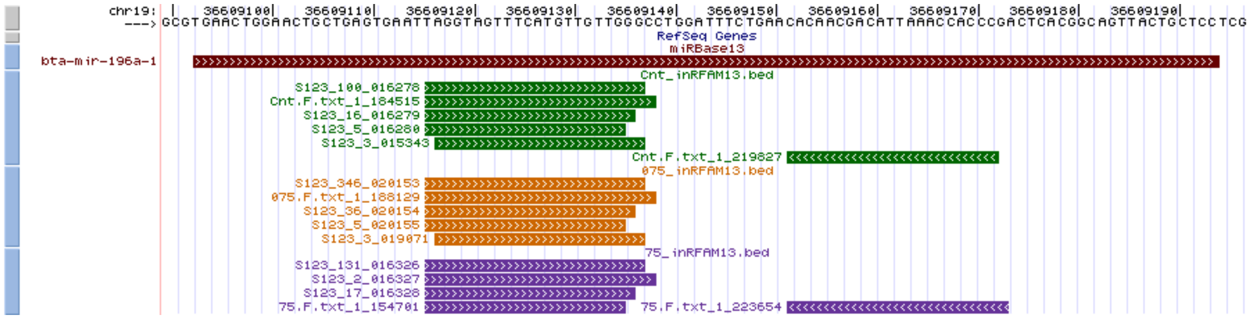

Glazov EA et al. Supplemental Figure S3

Fig S3f.

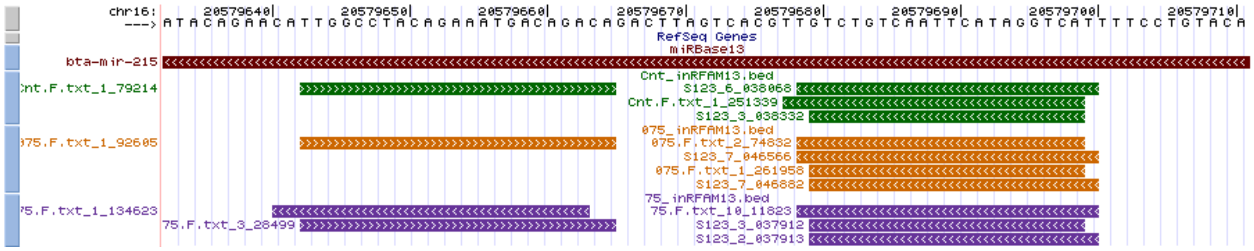

Fig S3g.

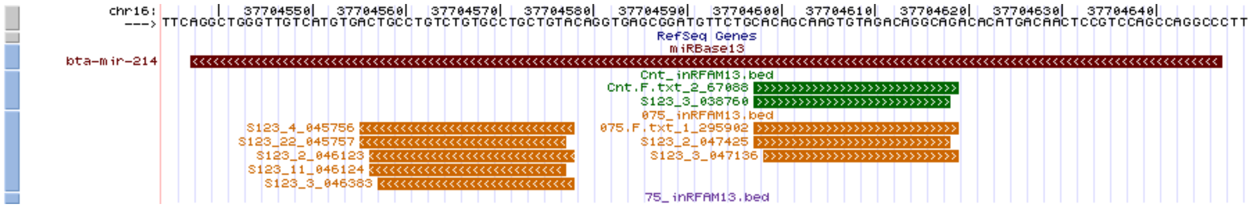

Fig S3h.

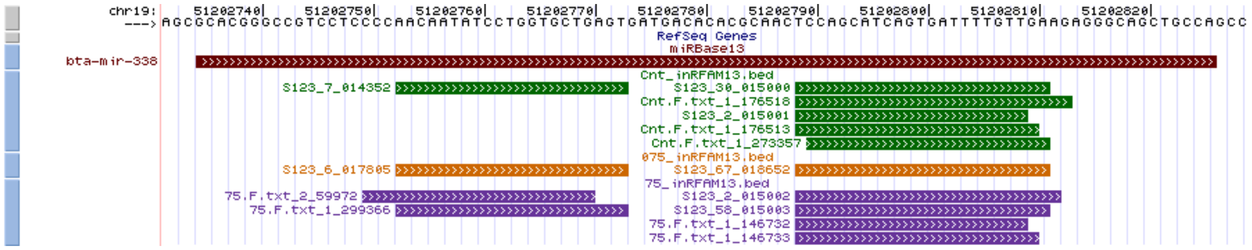

Supplemental Figures S3a-S3h. Bidirectional miRNA candidates. (S3a-S3d) New miRNA candidates that have evidence of transcription and predicted hairpin-like structures on both genomic strands. (**S3e-S3g)** Known miRNA candidates that have evidence of transcription and predicted hairpin-like structures on both genomic strands. (**S3h)** Cow miRNA bta-mir-338 that has evidence of bidirectional transcription in other species (Tyler et al. 2008). The figures shows UCSC genome browser screens displaying relative positions of predicted miRNA hairpin-like precursors (blue) or known miRNA precursor (deep red), and sequence tags originating from this loci in three small RNA libraries: mock-infected control - green, MOI 0.75 library - dark orange, MOI 7.5 library – magenta. Arrowheads indicate alignment of sequences relative to the genomic strands.
