# Supplementary material for: Repertoire of Bovine miRNA and miRNA-Like Small Regulatory RNAs Expressed upon Viral Infection
Source: PLoS One. 2009 Jul 27;4(7):e6349. doi: 10.1371/journal.pone.0006349 (PMC2713767; doi:10.1371/journal.pone.0006349)
Supplement: Figure S4 — Mirtrons (0.09 MB PDF) [file pone.0006349.s009.pdf]

Glazov EA et al. Supplemental Figure S4

Fig S4a.

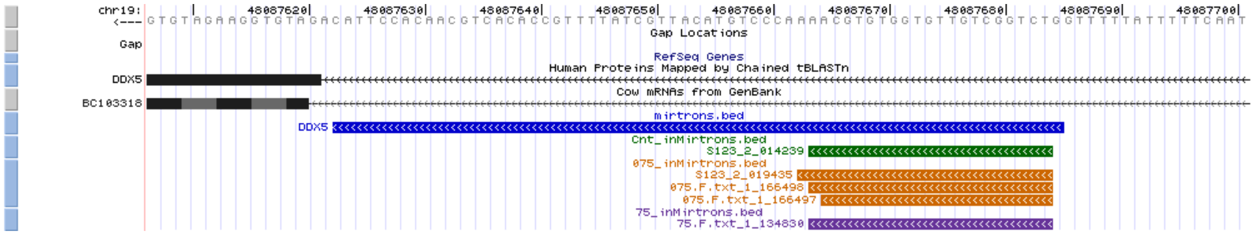

Fig S4b.

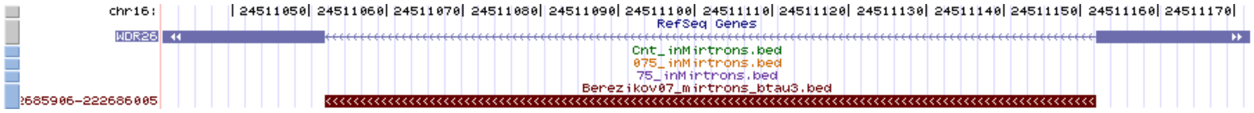

Fig S4c.

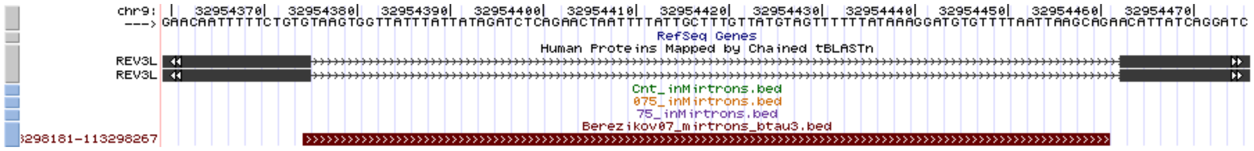

Fig S4d.

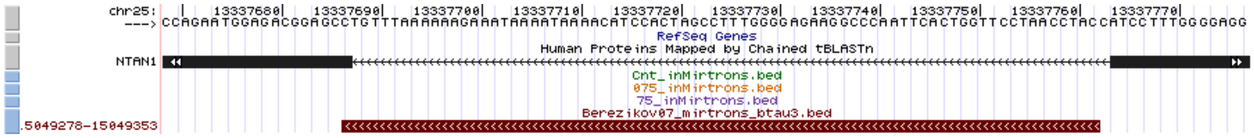

Fig S4e.

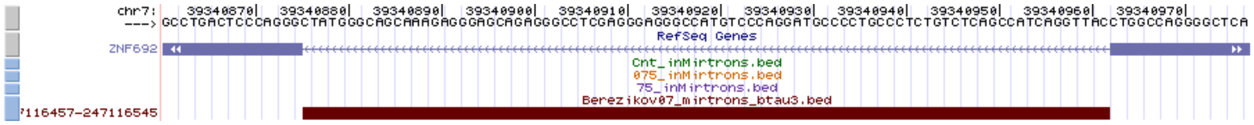

Supplemental Figures S4a-S4e. (S4a) Expressed mirtron localized within *DDX5* gene. **(S4b-S4e) Mirtron candidates.** Mirtron candidates that were identified based on sequence similarity and syntenic location in comparison to other mammalian genomes that didn't have evidence of expression in our small RNA libraries. The figures shows UCSC genome browser screens displaying relative positions of exon-intron splice junctions of RefSeq genes (top light blue) or human proteins mapped onto bovine genome (top black), predicted mirtrons (deep red).
