# Supplementary material for: Repertoire of Bovine miRNA and miRNA-Like Small Regulatory RNAs Expressed upon Viral Infection
Source: PLoS One. 2009 Jul 27;4(7):e6349. doi: 10.1371/journal.pone.0006349 (PMC2713767; doi:10.1371/journal.pone.0006349)
Supplement: Figure S5 — snoRNA-derived small RNAs (0.13 MB PDF) [file pone.0006349.s010.pdf]

Glazov EA et al. Supplemental Figure S5

Fig S5a.

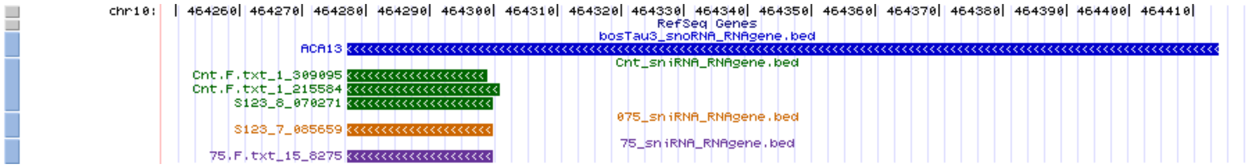

Fig S5b.

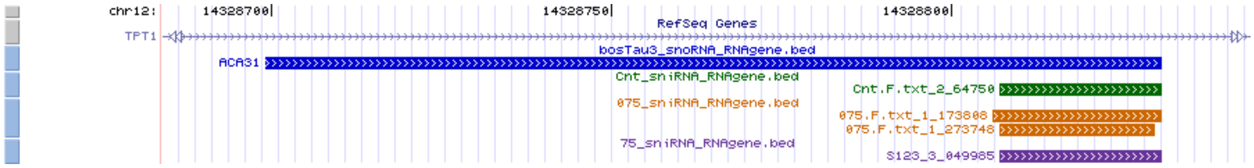

Fig S5c.

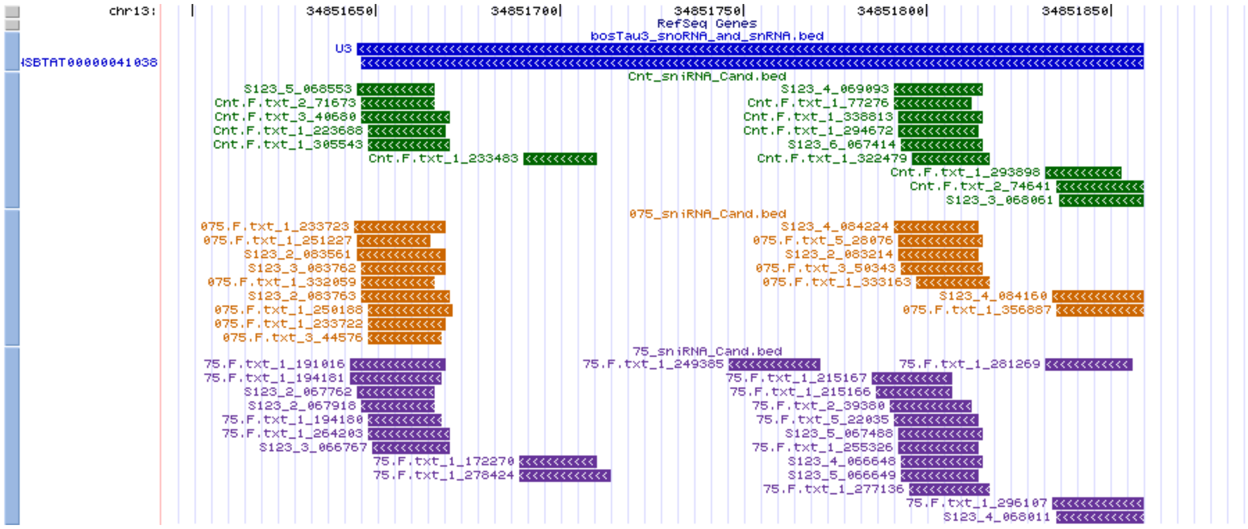

Fig S5d.

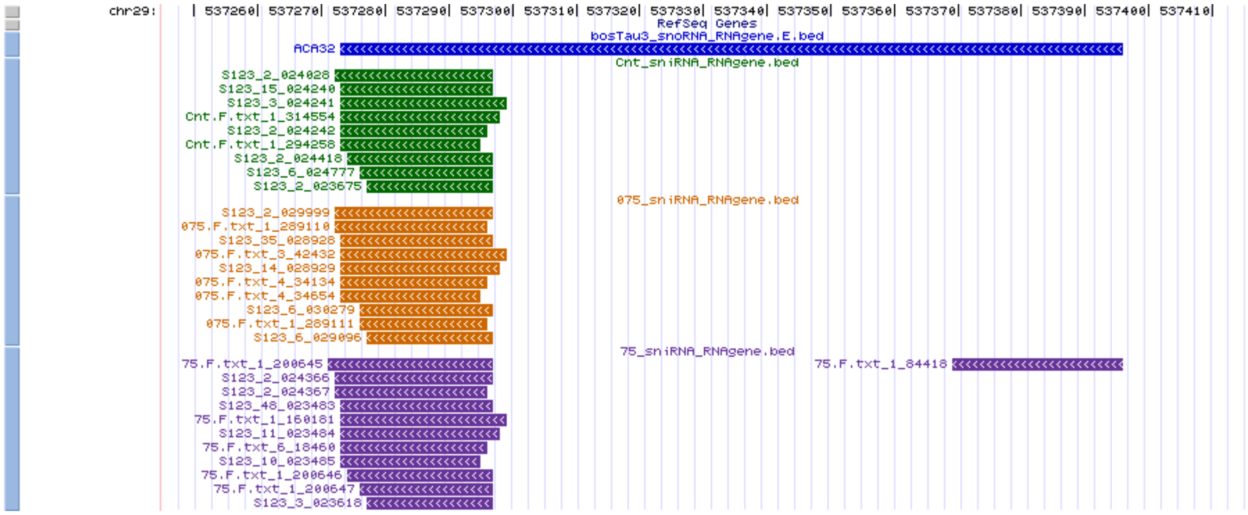

Glazov EA et al. Supplemental Figure S5

Fig S5e.

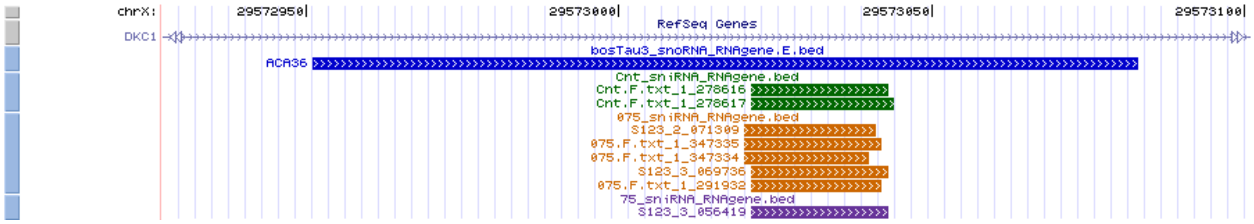

Fig S5f.

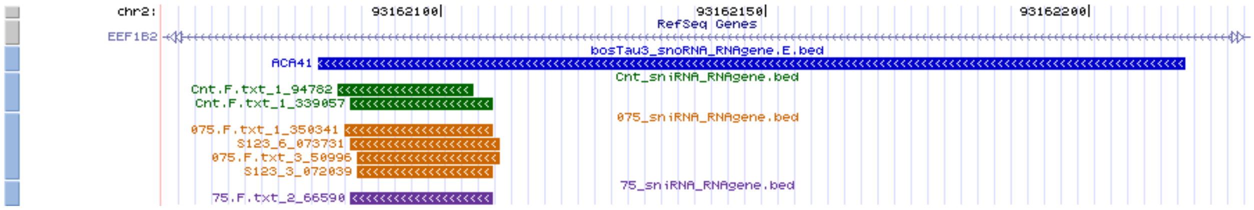

Fig S5g.

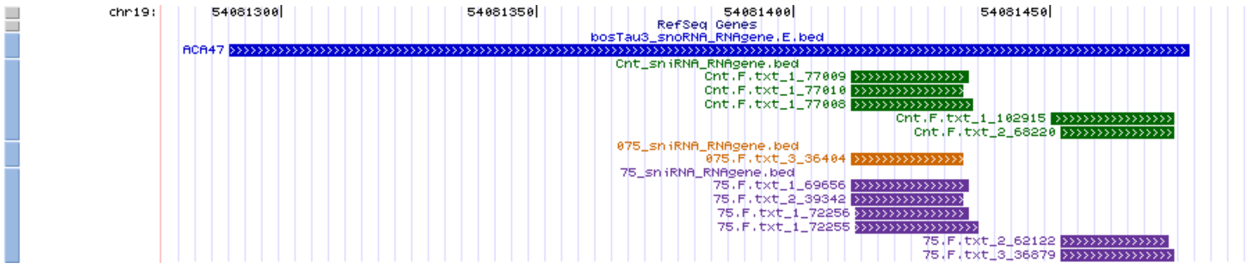

Fig S5h.

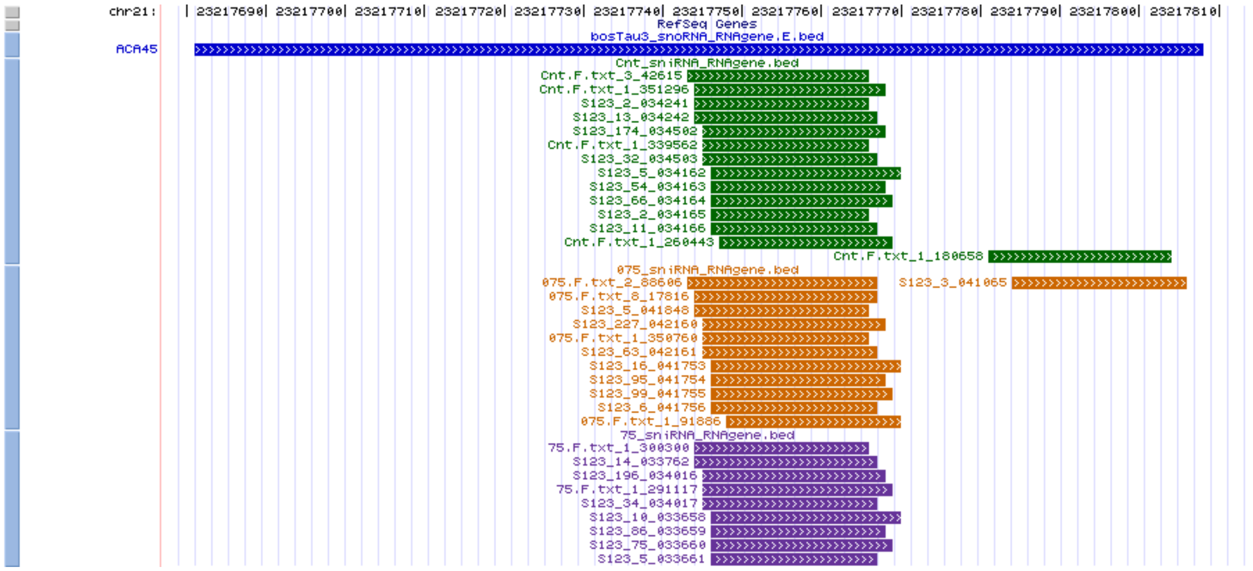

Fig S5i.

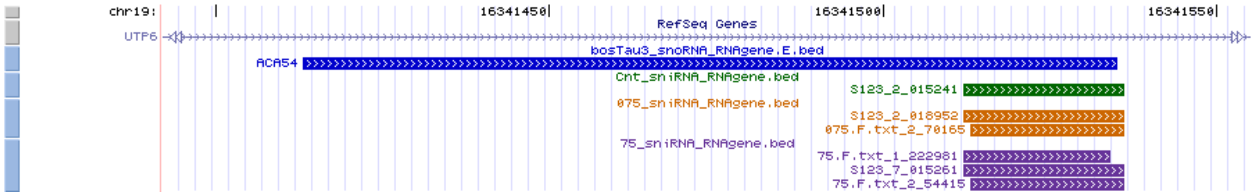

Glazov EA et al. Supplemental Figure S5

Fig S5j.

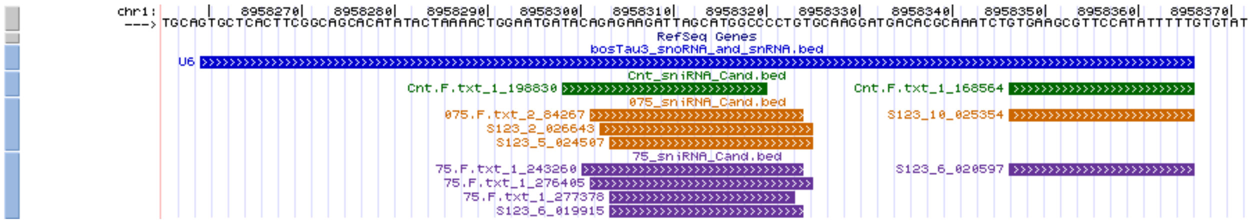

Fig S5k.

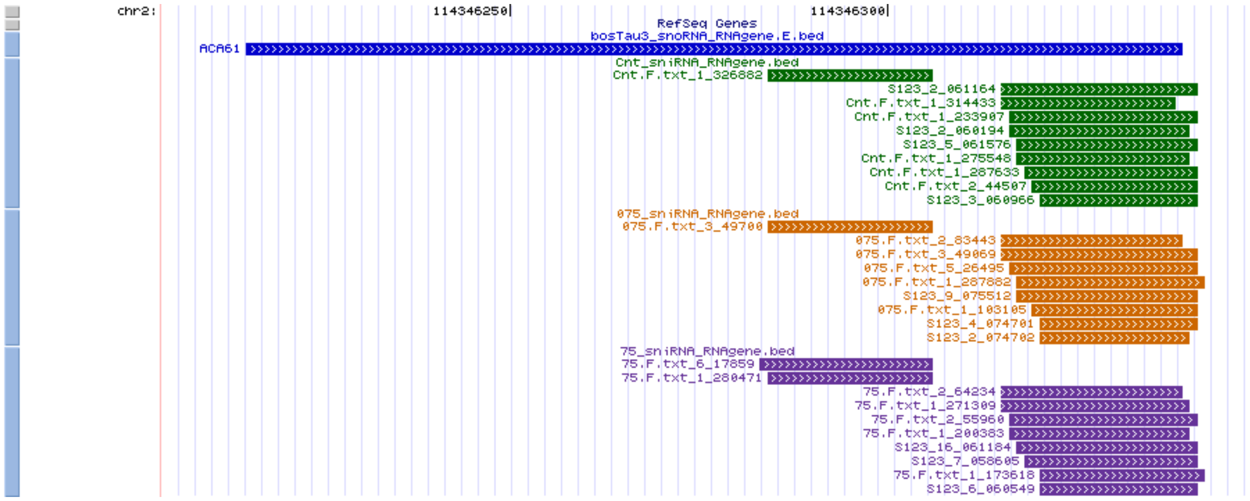

Supplemental Figures S5a-S5k. snoRNA-derived small RNAs. The figures shows UCSC genome browser screens displaying relative positions of bovine snoRNA or snRNA (blue) and sequence tags originating from this loci in three small RNA libraries: mock-infected control - green, MOI 0.75 library - dark orange, MOI 7.5 library – magenta. Arrowheads indicate alignment of sequences relative to the genomic strands.
