# Supplementary material for: Repertoire of Bovine miRNA and miRNA-Like Small Regulatory RNAs Expressed upon Viral Infection
Source: PLoS One. 2009 Jul 27;4(7):e6349. doi: 10.1371/journal.pone.0006349 (PMC2713767; doi:10.1371/journal.pone.0006349)
Supplement: Figure S6 — Bovine miRNA bta-mir-219 (0.06 MB PDF) [file pone.0006349.s011.pdf]

Glazov EA et al. Supplemental Figure S6

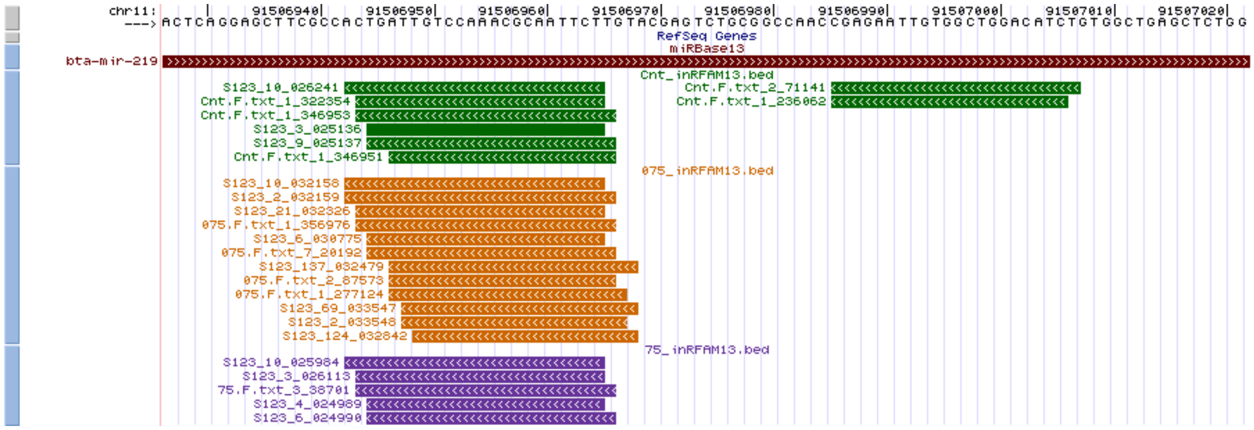

Supplemental Figure S6. Bovine miRNA bta-mir-219. Majority of small RNA tags originate from opposite genomic strand to annotated miRNA precursor. The figures shows UCSC genome browser screens displaying relative positions miRNA precursor (deep red) and sequence tags originating from this locus in three small RNA libraries: mock-infected control - green, MOI 0.75 library - dark orange, MOI 7.5 library – magenta. Arrowheads indicate alignment of sequences relative to the genomic strands.
