# Supplementary material for: Repertoire of Bovine miRNA and miRNA-Like Small Regulatory RNAs Expressed upon Viral Infection
Source: PLoS One. 2009 Jul 27;4(7):e6349. doi: 10.1371/journal.pone.0006349 (PMC2713767; doi:10.1371/journal.pone.0006349)
Supplement: File S1 — Supplemental methods. (0.03 MB PDF) [file pone.0006349.s012.pdf]

Supplemental methods

Virus and Cells.

The BoHV-1 virus utilised for this study was recovered using the infection clone, pBACBHV-37 [1], of the BoHV-1 strain V155 as previously described in Madin-Darby Bovine Kidney (MDBK) cells (ATCC number CCL-22) [2]. The MDBK cells were maintained in Earle's minimal essential medium containing non-essential amino acids, glutaMAX, 25 mM HEPES and 5% (v/v) foetal calf serum at 37oC in a 5% CO_2 atmosphere. All reagents utilised for cell and virus propagation were obtained from Invitrogen.

Preparation of RNA samples.

MDBK cells were seeded into flasks 24 h prior use and infected at approximately 80% confluency with BoHV-1 and incubated at 37 ^{o}C. The MDBK monolayers were infected at m.o.i. of either 0.75 or 7.5. At 6 h, 12, h or 24 h post-infection monolayers were harvested and total RNA recovered using Trizol reagent (Invitrogen) according to the manufacturer's instructions. Total RNA was recovered from mock infected MDBK monolayers for control purposes. The total RNA was resuspended in DEPC treated water. The RNA was quantified using a NanoDrop spectrophotometer and either used immediately or stored at -70 ^{o}C until required.

The small RNA fraction (<400 nucleotides) was recovered from total RNA using previously described methods [3]. Briefly, polyethylene glycol 8000 and NaCl were added to the total RNA solution and the volume adjusted to give final concentrations of 10% (w/v) and 500 mM respectively, followed by incubation on ice for 30 min. The samples were centrifuged at 10,000 g for 10 min at 4 ^{o}C. The supernatant was transferred to a new tube and 3 volumes of 100% ethanol added. The small RNA was allowed to precipitate by incubation overnight at -20 ^{o}C prior to recovery by centrifugation at 10,000 g at 4 ^{o}C for 30 min. The resultant RNA pellet was washed once with 75% ethanol and air-dried. The small RNA pellet was resuspended in de-ionised formamide for electrophoresis prior to Northern blotting analyses. The concentration and quality of the small RNA fractions were estimated using either a NanoDrop Spectrophotometer or gel electrophoresis.

Analysis of sequencing data

Sequencing libraries and individual sequence reads with the base quality scores were produced by GeneWorks Pty Ltd using Illimina Genome Analyzer 1 instrument and software. The identical sequence reads were counted to produce a 'read count' score. All duplicated sequences were eliminated from the initial dataset to produce a non-redundant set of the unique sequences, hereafter referred to as sequence tags. After trimming the 3' adaptor sequence (5'-TCGTATGCCGTCTTCTGCTTG-3') sequence tags were mapped onto cow genome assembly bosTau3 (August 2006), using BLAT software [4] with the following parameters: -noHead -minMatch=1 -oneOff=1 -minIdentity=100 -tileSize=12. All partial alignments and alignments containing gaps were eliminated from further analyses.

To identify sequence tags originating from coding exons, repeats, rRNA, tRNA, snRNA and snoRNA we used UCSC "RefGene", "RepeatMasker" and NCBI "RefSeq" data [5], as well as our own set of ncRNA annotations compiled from the NCBI GenBank data (http://www.ncbi.nlm.nih.gov/) Sequence tags matching parts of cow rRNA and tRNA sequences were recorded and excluded from further analyses.

For the analysis of small RNAs originating from snRNA and snoRNA loci we mapped curated sets of human snoRNA annotations obtained through the RNA Gene UCSC Genome Browser track (rnaGene) [5] using the UCSC syntenic alignment utility, liftOver, requiring 95% sequence identity.

For RNA structure prediction the genomic coordinates of the sequence tags were extended by 60 nt at their 5' and 3' ends, and the corresponding genomic sequences were retrieved and used as an input for the RNAfold software [6]. To identify potential miRNA precursors in the set of the predicted RNA structures we applied a filtering mechanism based on sequence, structural features, and minimum free energy of the known vertebrate miRNA precursors present in miRBase [7]. In brief, we eliminated all predicted hairpin-like structures that were shorter than 53 nt in length, or had either of the following: a terminal loop larger than 22 nt, bulges larger than 18 nt, runs of more than 7 'Gs', runs of more than 8 'Cs', runs of more than 13 'As', runs of more than 11 'U/Ts', or had a minimum free energy above -15 kcal/mol. In addition, all remaining pre-miRNA candidates were analyzed for distribution of the sequence tags to ensure that the RNA hairpin structure and positions of sequence tags meet basic requirements of the Drosha/Dicer pre-miRNA processing complex.

Analysis of the evolutionary conservation of the newly identified miRNAs

The sequences of the predicted RNA hairpin structures identified as pre-miRNA candidates were used to search human (hg18), dog (canFam2), opossum (monDom4) and horse (equCab2) genomes using BLAT [4] with the following parameters: -noHead -minMatch=1 -oneOff=1 -minIdentity=90 -tileSize=8. Sequence alignments covering at least 90% of the length of the bovine pre-miRNA were considered as potential orthologs and used in further RNA secondary structure analyses. Orthologous candidate sequences identified in human, dog, opossum, and horse were used to predict RNA secondary structure as described above. New bovine miRNA sequences that did not have detectable orthologous sequences in any of the analyzed mammalian genomes using the described method, were considered as potential ruminant-specific miRNAs.

Analysis of the relative abundance of miRNAs in three RNA libraries

Similar to credibility interval approaches (as opposed to hypothesis testing) reported for the analysis of SAGE data [8], we employed a semi-parametric approach to identify miRNAs showing statistically significant difference in relative abundance (as reflected by total count of individual sequence reads) between the three small RNA libraries. Firstly, for each sample, the abundance of each miRNA was expressed in reads per million (rpm) and this value was log-transformed to approximate a

Gaussian distribution. Secondly, for each miRNA, the observed range in log-transformed rpm value across the three samples was recorded. Finally, miRNA with range values lying beyond three standard errors of the mean of ranges were deemed to be significantly different between the samples at $P < 0.01$.

Additional references

1. Mahony TJ, McCarthy FM, Gravel JL, West L, Young PL (2002) Construction and manipulation of an infectious clone of the bovine herpesvirus 1 genome maintained as a bacterial artificial chromosome. J Virol 76: 6660-6668.
2. Madin SH, Darby NB, Jr. (1958) Established kidney cell lines of normal adult bovine and ovine origin. Proc Soc Exp Biol Med 98: 574-576.
3. Mitter N, Sulistyowati E, Dietzgen RG (2003) Cucumber mosaic virus infection transiently breaks dsRNA-induced transgenic immunity to Potato virus Y in tobacco. Mol Plant Microbe Interact 16: 936-944.
4. Kent WJ (2002) BLAT--the BLAST-like alignment tool. Genome Res 12: 656-664.
5. Karolchik D, Kuhn RM, Baertsch R, Barber GP, Clawson H, et al. (2008) The UCSC Genome Browser Database: 2008 update. Nucleic Acids Res 36: D773-779.
6. Hofacker IL (2003) Vienna RNA secondary structure server. Nucleic Acids Res 31: 3429-3431.
7. Griffiths-Jones S, Grocock RJ, van Dongen S, Bateman A, Enright AJ (2006) miRBase: microRNA sequences, targets and gene nomenclature. Nucleic Acids Research 34: D140-D144.
8. Vencio RZ, Brentani H, Pereira CA (2003) Using credibility intervals instead of hypothesis tests in SAGE analysis. Bioinformatics 19: 2461-2464.
